# Definitions and symptoms of the post-COVID syndrome: an updated systematic umbrella review

**DOI:** 10.1007/s00406-024-01868-y

**Published:** 2024-07-25

**Authors:** Julian Gutzeit, M. Weiß, C. Nürnberger, C. Lemhöfer, K. S. Appel, E. Pracht, J.-P. Reese, C. Lehmann, M. C. Polidori, G. Hein, J. Deckert

**Affiliations:** 1 Center of Mental Health, Department of Psychiatry, Psychosomatic and Psychotherapy, University Hospital Würzburg, Margarete-Höppel-Platz 1, 97080 Würzburg, Germany; 2 Department of Psychology III - Psychological Methods, Cognition, and Applied Research, Julius-Maximilians-Universität Würzburg, Röntgenring 11, 97070 Würzburg, Germany; 3 Department of Psychology I - Biological Psychology, Clinical Psychology and Psychotherapy, Julius-Maximilians-Universität Würzburg, Marcusstraße 11, 97070 Würzburg, Germany; 4 Institute of Clinical Epidemiology and Biometry, University of Würzburg, Josef-Schneider-Straße 2, 97080 Würzburg, Germany; 5 Institute for Physical and Rehabilitative Medicine, University Hospital Jena, Jena, Germany; 6 Center for Internal Medicine, Medical Department 2 (Hematology/Oncology and Infectious Diseases), Goethe University Frankfurt, University Hospital, Frankfurt, Germany; 7 Faculty of Medicine and University Hospital Cologne, Department I for Internal Medicine, University of Cologne, Cologne, Germany; 8 German Center for Infection Research, Partner site Bonn-Cologne, Cologne, Germany; 9 Center for Molecular Medicine Cologne (CMMC), University of Cologne, Cologne, Germany; 10 Aging Clinical Research, Department II of Internal Medicine, Center for Molecular Medicine Cologne, Faculty of Medicine, University Hospital Cologne, University of Cologne, Herderstraße 52, 50931 Cologne, Germany; 11 Cologne Excellence Cluster on Cellular Stress- Responses in Aging- Associated Diseases (CECAD), Faculty of Medicine, University of Cologne, University Hospital Cologne, Cologne, Germany

**Keywords:** Post-COVID syndrome (PCS), Long COVID, Fatigue, Neurological complaints, Diagnostic criteria, Systematic review

## Abstract

Post-COVID syndrome (PCS) describes a persistent complex of symptoms following a COVID-19 episode, lasting at least 4 to 12 weeks, depending on the specific criteria used for its definition. It is often associated with moderate to severe impairments of daily life and represents a major burden for many people worldwide. However, especially during the first two years of the COVID-19 pandemic, therapeutic and diagnostic uncertainties were prominent due to the novelty of the disease and non-specific definitions that overlooked functional deficits and lacked objective assessment. The present work comprehensively examines the status of PCS definitions as depicted in recent reviews and meta-analyses, alongside exploring associated symptoms and functional impairments. We searched the database Pubmed for reviews and meta-analysis evaluating PCS in the period between May 31, 2022, to December 31, 2023. Out of 95 studies, 33 were selected for inclusion in our analyses. Furthermore, we extended upon prior research by systematically recording the symptoms linked with PCS as identified in the studies. We found that fatigue, neurological complaints, and exercise intolerance were the most frequently reported symptoms. In conclusion, over the past eighteen months, there has been a notable increase in quantity and quality of research studies on PCS. However, there still remains a clear need for improvement, particularly with regard to the definition of the symptoms necessary for diagnosing this syndrome. Enhancing this aspect will render future research more comparable and precise, thereby advancing and understanding PCS.

**Supplementary Information:**

The online version contains supplementary material available at 10.1007/s00406-024-01868-y.

## Introduction

First identified in Wuhan, China, in 2019, SARS-CoV-2 led to the global COVID-19 pandemic, with around 775 million cases and over 7 million deaths by 2023 [1], and is now evolving into an endemic disease [2]. This is particularly problematic because SARS-CoV-2 infections are frequently associated with prolonged effects after COVID-19. This syndrome is referred to as long-COVID, post-COVID, or post-COVID syndrome [PCS; 3]. Although PCS is difficult to clearly diagnose due to varying definitions and temporal criteria [4], some studies estimated that 54% of hospitalized and 34% of non-hospitalized COVID-19 patients suffer from PCS [5], with a combined prevalence of 43% [6], while other studies more conservatively estimate the prevalence to be 4% and 5% among three-times vaccinated infected adults [7, 8]. Thus, even though the international public health emergency declared by the World Health Organization (WHO) has ended [9], the impact of COVID-19 and PCS is still a significant burden for millions of patients worldwide and needs to be more thoroughly investigated.

Lemhöfer et al. [4] investigated the characteristics of PCS in a narrative umbrella review. They found strong heterogeneity in the definitions of PCS across the included studies. Only 11 out of 16 included studies provided a defined framework for PCS, with just 58% incorporating follow-up analyses of ≥ 12 weeks as required by the WHO [10] and the UK National Institute for Health Care Excellence [11] definitions (for an overview of specific definitions, see Table 1). Even though functional aspects, in the context of the biopsychosocial model, might contribute to ongoing limitations in respiration and exertion, particularly in individuals with a mild acute course, only a minority of 2 included studies addressed functional impairments and the quality of life. The authors of the review criticize the insufficient representation of functional perspectives. Within this context, in addition, the influence of chronological age, frailty and preexisting physiological vulnerability has been poorly investigated so far. Accordingly, newer studies suggests that functional impairments play a vital role in the diagnostics and treatment of PCS and should thus be considered carefully in research [12, 13]. Another criticism by Lemhöfer et al. is the inadequate description of the symptoms that need to be present to diagnose PCS. According to Peter et al. [14], fatigue and cognitive deficits are the symptoms most common in patients with PCS. However, the WHO and NICE definitions do not require these symptoms for diagnosis. Finally, the authors underscore the importance of a standardized assessment protocols for PCS, especially in large cohort studies such as the German National Pandemic Cohort Network (NAPKON) of the Network University Medicine [NUM; 15].

**Table 1:** Overview of different definitions of PCS from different health organizations

Organization	Definition
World Health Organization (WHO)	**Adults**: The development of new symptoms 3 months after the initial SARS-CoV-2 infection, with these symptoms lasting for at least 2 months with no other explanation [16]
	**Children**: Post-COVID-19 condition occurs in young people with a history of confirmed SARS-CoV-2 infection, with at least one persisting physical symptom for a minimum duration of 12 weeks after initial testing that cannot be explained by an alternative diagnosis. The symptoms have an impact on everyday functioning, may continue or develop after COVID infection, and may fluctuate or relapse over time. The positive COVID-19 test referred to in this definition can be a lateral flow antigen test, a PCR test or an antibody test. [17]
National Institute for Health Care Excellence (NICE)	Signs and symptoms that develop during or after an infection consistent with COVID‑19, continue for more than 12 weeks and are not explained by an alternative diagnosis [11, p5]
National Institute of Health (NIH)	Health problems that some people experience a few months after a COVID-19 diagnosis. Symptoms of Long COVID may be the same as or different from symptoms of COVID-19. [18]
Centers for Disease Control (CDC)	A vast range of ongoing health problems (e.g., cardiovascular, respiratory, and neuropsychiatric symptoms) that can last for > 4 weeks after an individual has been infected by SARS-CoV-2 virus. [19]

The aim of this study is to provide an updated review following the work by Lemhöfer et al. (2022). With more than one and a half years elapsed since their publication, considerable relevant studies and scientific progress have emerged. This umbrella review attempts to assess whether recent research has converged on clearer definitions of PCS and whether functional impairments have received more recognition in recent research. We also extend the analyses of Lemhöfer et al. (2022) by systematically investigating the symptoms associated with PCS in these studies. Furthermore, all definitions of PCS share the requirement of a prior confirmed SARS-CoV-2 infection for diagnosis. Therefore, we assess whether and how the SARS-CoV-2 infection is diagnostically confirmed in patients in current research. This study is based on published literature. All data supporting the findings of this study are available within the paper and its Supplementary Information. All primary sources cited in the table are publicly available through their original publications. No new raw data were created for this study.

## Methods

### Search strategy and information sources

We performed a systematic electronic literature search in the Pubmed database. To update the work of Lemhöfer et al. [4] we searched for articles that were published between May 31 2022 and December 31 2023, seamlessly following the temporal inclusion criteria of Lemhöfer et al. We used the same search algorithm, searching for ((Post-COVID) OR (Long-COVID) OR (Long-haulers)) AND ((Deficits) OR (Sequale) OR (Consequences) OR (Outcome) OR (Impact)) (all as text words) and filtered for systematic reviews.

### Eligibility

Studies were included if they met the following criteria:

1. Language: studies written in English or German.
2. Study type: systematic reviews.
3. Population: reviews including studies with post-COVID patients.
4. Outcome: studies reporting symptoms of PCS in at least two distinct domains (e.g., neurological symptoms, fatigue, cardiological symptoms).

### Study selection and data extraction

Two reviewers (JG, MW) independently screened the titles and abstracts to exclude studies that did not meet the inclusion criteria. Disagreements were resolved by discussion.

### Analyses

The included studies were analyzed regarding their definition of PCS, temporal inclusion criteria, and reporting of functional impairments [following 4]. Additionally, studies were analyzed for reported PCS symptoms. The following categories were defined: chemosensory deficits, fatigue, exercise intolerance, joint and/or muscle pain, ear-nose-throat ailments, pulmonary symptoms, cardiac symptoms, gastrointestinal ailments, neurological ailments, dermatological ailments, infection signs, sleep disturbance [following 13], psychiatric symptoms (such as anxiety, depressive mood, etc.), renal symptoms (relating to the filtration and production of urine, involving the kidneys), and urinary symptoms (involving the bladder and urethra) [20]. Each review was coded as either reporting symptoms of the categories above or as not reporting such symptoms. We then calculated the proportions of all papers reporting these symptoms. In the same way, we analyzed which papers reported PCS-related impairments of quality of life, and whether and how SARS-CoV2 infection was confirmed in patients.

## Results

**Fig. 1:**
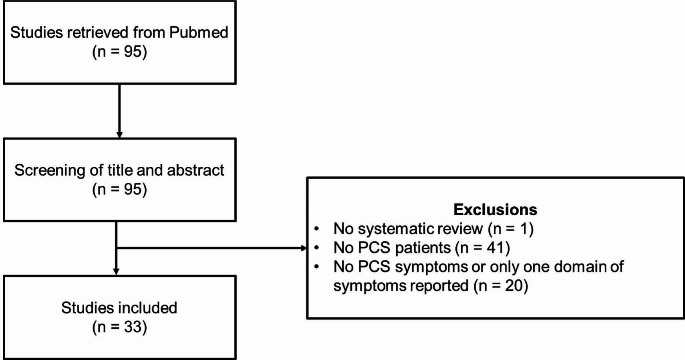
Flow chart of study selection process according to eligibility criteria

Our initial search identified 95 studies, of which one was excluded for not being a systematic review, 41 studies for nut focusing on PCS patients, and 20 studies did not report on PCS symptoms or only reported a specific pre-defined symptom (e.g., anosmia, ageusia, etc.). This resulted in 33 studies included in the present review [21–53]. The study selection process is illustrated in Fig. 1. and a summary of all included studies can be found in Table 2.

### Definition of PCS, temporal inclusion criteria, and SARS-CoV-2 confirmation

**Fig. 2:**
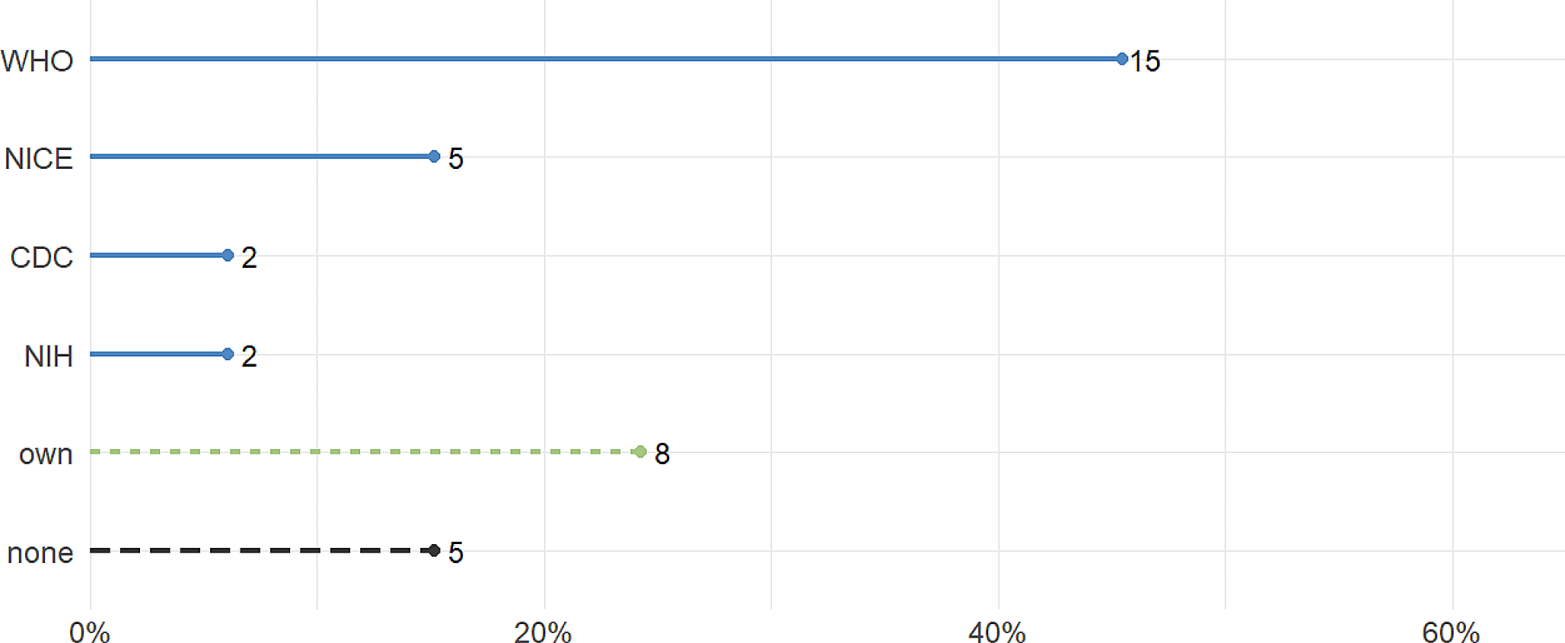
Proportions and absolute values (number next to point) of used definitions in each study. Some studies used multiple definitions. WHO = World Health Organization, NICE = National Institute for Health and Care Excellence, CDC = Centers for Disease Control and Prevention, NIH = National Institutes of Health

Among the studies analyzed, a substantial majority, accounting for 85% (*n* = 28), furnished a clear definition of PCS. The most frequently used definition was the WHO definition, used by 45% (*n* = 15) studies. Additionally, 15% (*n* = 5) used the NICE definition, while 6% of studies (*n* = 2) either used the US Centers for Disease Control and Prevention (CDC), or the US National Institute of Health (NIH) definition, respectively. Only one study used the Office for National Statistics (UK) definition, and one study the National Research Action Plan on Long-COVID (US) definition. Approximately a quarter of the studies, comprising 24% (*n* = 8) used their own definition of PCS (see Fig. 2). It should be noted that some studies used multiple definitions simultaneously. For an overview of the specific definitions, please refer to Table 1.

Among the studies analyzed, 30% (*n* = 10) of all studies included patients examined 4 weeks after the SARS-CoV-2 infection. A slightly lower proportion, 27% (*n* = 9) followed the WHO and NICE temporal criterion for PCS and included patients 12 weeks or more after SARS-CoV-2 infection. Furthermore, 12% (*n* = 4) of all studies applied different temporal criteria, ranging from 3 weeks to 2 years follow-up. Notably, 27% (*n* = 9) had no temporal inclusion criterion for patients. An overview of all definitions and temporal inclusion criteria used in the included studies is shown in Table 2.

The most common method of confirming SARS-CoV-2 infections in patients was PCR testing, reported by 46% (*n* = 15) of all included studies, followed by antibody tests (33%, *n* = 11) and antigen tests (21%, *n* = 7). In 18% (*n* = 6) of the studies, infections were confirmed by clinicians assessing clinical symptoms. Additionally, 12% (*n* = 4) of the studies reported unspecified ‘laboratory diagnostics’ as the criterion for infection confirmation. Both radiological diagnosis and self-reported symptoms were reported in 6% of the studies (*n* = 2). Furthermore, 15% of the studies (*n* = 5) reported that SARS-CoV-2 infections were confirmed in patients, but they did not further specify the methods used. Lastly, 30% of all studies (*n* = 10) did not address the necessity of confirming the SARS-CoV-2 infection or did not report any confirmation methods. A detailed overview of SARS-CoV-2 confirmation methods in each study can be found in supplementary Table S1.

**Table 2:** Characteristics of included reviews and meta-analyses (*n* = 33)

Reference	Included studies (*n*)	Age	Definition	Temporal inclusion criterion	Assessed functional impairments
Baroni [34]	*not reported*	*not reported*	National Research Action Plan on Long-COVID (US)	symptoms 4 weeks after infection	general impairment of quality of life
Behnood [53]	17	mean age 4-15 range 0–19	none	none	none
Bertuccelli [25]	25	mean age 43-79 range 26–86	own	symptoms 12 weeks after infection	cognitive impairments
Chandan [38]	5	mean age 18-57 range 18–75	WHO	none	functional physical capacity
Di Gennaro [42]	196	mean age 52	WHO	symptoms persisting for 8 weeks within 12 weeks of infection	nonspecific
Espinoza [50]	22	mean age 42-79 range 17–87	WHO, NICE	none	cognitive dysfunctions
Gao [26]	18	mean age 41-67 range 12–70	WHO	symptoms persisting for 8 weeks within 12 weeks of infection	none
Hallek [33]	716 *(screened RCTs)*	*not reported*	WHO	symptoms persisting for 8 weeks within 12 weeks of infection	psychosocial consequences, loneliness
Hirt [46]	6	< 18 years	NICE, WHO	symptoms ≥ 3 weeks after infection	none
Hoshijima [52]	21	*not reported*	NIH, WHO	symptoms ≥ 2 months after diagnosis or ≥ 1 month after recovery	none
Hossain [47]	12	range 18–82	own	none	cognitive deficits, impairment of everyday well-being, need for rehabilitation and return to work, social isolation
Houben [23]	27	mean age 31-71 range 13–80	WHO	symptoms persisting for 8 weeks within 12 weeks of infection	cognitive restrictions
Kelly [43]	60	range 15–90	NICE	symptoms 4 weeks after infection	none
Kerzhner [49]	26	mean age 38-70 range 10–88	NIH, WHO	none	cognitive impairments, movement impairments
Korchut [48]	45	> 18 years	own	symptoms 4 weeks after infection	limited mobility, cognitive restrictions, walking impairments, limitations due to dyspnea
Marchi [22]	33	mean age 34–73	own	symptoms 4 weeks after infection	psychiatric symptoms
Marra [45]	6	*not reported*	CDC	symptoms ≥ 3 weeks after infection	cognitive impairments
Notarte [44]	17	median age 34-68 range 0–73	none	none	cognitive impairments, impairments in social roles
O’Mahoney [36]	184	median age 3–74	own	symptoms 4 weeks after infection	impairment of usual activities
Paterson [28]	18	mean age 5-87 range 1–93	none	symptoms 4 weeks after infection	general functional impairments, QOL, participation in the labor market, psychological stress, cognitive impairments
Pellegrino [29]	22	median age 9-18 range 4–18	NICE, CDC, WHO (children)	symptoms 4 weeks after infection	impairment of everyday skills
Perrottelli [32]	72	mean age 20-70 range 18–96	none	symptoms 12 weeks after infection	cognitive impairments
Pillay [31]	17	median age 34–70	WHO	symptoms 12 weeks after infection	nonspecific
Pouliopoulou [51]	14	mean age 41–68	WHO	none	functional exercise capacity, dyspnea, and quality of life
Rahmati [21]	12	median age 40–61	own	at least 2 years follow-up	physical impairments
Salari [30]	52	mean age 9–73	none	symptoms 4 weeks after infection	fatigue
Sobrino-Relaño [35]	25 *(included in review)* 6 *(included in meta-analysis)*	mean age 37–67	WHO	infection ≥ 3 weeks ago	neuropsychological deficits
Teodoro [41]	102	mean/median age 40–59	none	none	functional neurological disorders
Veronese [24]	2	mean age 42 range 19–49	WHO	symptoms 12 weeks after infection	none
Watanabe [39]	6	mean age 40–54	own	symptoms 4 weeks after infection	none
Yang [27]	72	median age 32-71 range 25–90	WHO	symptoms 12 weeks after infection	cognitive dysfunctions, sexual dysfunctions
Zakia [40]	23	median age 39–68	NICE	symptoms 4 weeks after infection	psychiatric symptoms
Zheng [37]	17	mean age 34-56 range 11–80	own	none	none

Note. Age is reported as the range between the median or mean ages from the included studies and, where applicable, as the range from the youngest to the oldest participant. CDC = Centers for Disease Control (US), NICE = National Institute for Health and Care Excellence (UK), NIH = National Institute of Health (US), RCT = Randomized Controlled Trial, QOL = Quality of Life, WHO = World Health Organization

### Symptoms, quality of life, functional assessments, and age

**Fig. 3:**
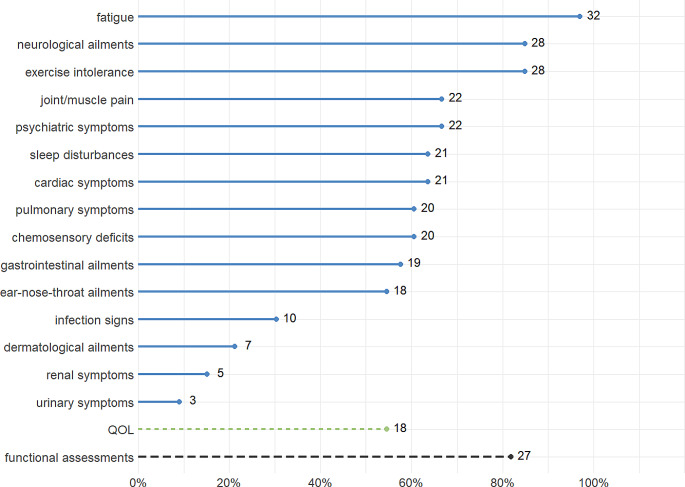
Proportions and absolute values (number next to point) of studies reporting on each symptom, on quality of life (QOL), and on functional assessments

The number of studies reporting each symptom, quality of life, and functional assessments are illustrated in Fig. 3. The most commonly reported symptoms were fatigue, reported by 97% of all included studies (*n* = 32), neurological ailments, reported by 85% (*n* = 28) of the studies, and exercise intolerance, reported by 82% (*n* = 28) of all studies. Most of the included reviews reported joint and/or muscle pain (67%; *n* = 22), psychiatric symptoms (67%; *n* = 22), sleep disturbances (67%; *n* = 22), cardiac symptoms (64%; *n* = 21), pulmonary symptoms (61%; *n* = 20), chemosensory deficits (61%; *n* = 20), gastrointestinal ailments (58%; *n* = 19), and ear-nose-throat ailments (55%; *n* = 18). Less frequently investigated symptoms included infection signs (30%; *n* = 10) and dermatological ailments (21%, *n* = 7). Only a small minority of papers reported renal symptoms (15%; *n* = 5), and/or urinary symptoms (9%; *n* = 3).

Quality of life (QOL) considerations were addressed in slightly more than half of the included studies (55%; *n* = 18). Among these studies, the analysis of QOL assessments across studies, the 36-Item Short Form Health Survey (SF-36) was the most frequently reported tool, used in 15% (*n* = 5) of the cases. The Neurology Quality of Life (Neuro-QoL), 12-Item Short Form Health Survey (SF-12), Patient-Reported Outcomes Measurement Information System (PROMIS), EuroQol Visual Analogue Scale (EQ-VAS), St. George’s Respiratory Questionnaire (SGRQ), and EuroQol five dimensions questionnaire with five levels (EQ-5D-5 L) followed closely, each being reported in 6% of all studies (*n* = 2). The World Health Organization Quality of Life (WHOQOL), Pediatric Quality of Life Inventory, and qualitative methods were each reported in 3% of the studies, (*n* = 1, respectively). In total, only 8 of all studies specified which QoL assessments were used, accounting for 44% of studies reporting on QoL and 24% of all studies. Functional assessments were reported in 82% (*n* = 27) of all studies. Cognitive impairments were most frequently described (33%, *n* = 11). An overview of all reported functional impairments is shown in Table 2. 91% (*n* = 30) of the studies report the ages of participants, with an overall age range from 0 to 90 years. 76% (*n* = 25) provide the mean or median ages of the sample sizes of the included studies. Among all studies, 33% (*n* = 11) included children or adolescents (under 18 years), and 64% (*n* = 21) report the inclusion of patients over 60 years. For an overview over reported symptoms, QOL, functional assessments, and age see Fig. 3.

A detailed summary of all symptoms reported in each study along QOL assessments is given in supplementary Table S1.

## Discussion

This study provides a refined review, expanding upon the basis laid by Lemhöfer et al. (2022), who focused on review articles and meta-analyses related to PCS. Their comprehensive literature search included studies available until May 31, 2022. Since then, there has been a significant increase in the number of reviews published and research in the field has advanced considerably. Lemhöfer et al. identified 52 studies, from which they included 16 in their analysis. Using the same search algorithm and inclusion criteria in the 1.5 years following their study, we identified 95 studies. Notably, we included more than double the number of studies in our analysis compared to their effort. This substantial increase in the number of relevant studies indicates significant progress in PCS research.

In their original study, Lemhöfer et al. criticized the heterogeneity and inadequacy of PCS definitions. They reported that only 11 of the 16 included studies (69%) provided a definition of the PCS, which was affirmed by similar findings in more recent reviews [54, 55]. Compared to this rate, we found a considerable improvement in quality, with 85% of all included studies providing at least some definition of the PCS. However, there was still no consensus on which definition was preferred. The predominant choice among studies in this umbrella review was either the WHO or the NICE definition, or a combination of both (see Table 1 for specific definitions). Based on their widespread application, we recommend using either the WHO or NICE definition for future research and diagnosis. Both definitions share a temporal scope of 12 weeks, differing only in the specific duration of symptom persistence (WHO requires at least 8 weeks, NICE at least 12 weeks). Despite this discrepancy, both definitions are expected to yield the same diagnosis after one more month of symptom persistence, demonstrating significant overlap.

An additional significant challenge in reaching a diagnostic consensus is the complex and heterogeneous terminology used to describe different SARS-CoV-2 sequelae. According to a recent comment on consensus definitions for long COVID, Munblit and colleagues [56] provided the following overview: *Long COVID*, proposed by patient-researchers, “can be broadly defined as signs, symptoms, and sequelae that continue or develop after acute COVID-19 or SARS-CoV-2 infection for any period of time” [for overview, see 57]. The term *persistent symptoms or COVID-19 consequences*, commonly used in research, refers to “persistent signs and symptoms that continue or develop after acute COVID-19 for any period of time” [58]. The definitions referenced in the present study further vary in their specific terminology. The WHO uses the term *post-COVID-19 condition*, while NICE refers to this syndrome as *post-COVID-19 syndrome*, in distinction to *long COVID*, which is defined as “signs and symptoms of COVID-19 from 4 weeks up to 12 weeks” [11]. The CDC uses both *long COVID* and *post-COVID*, and the NIH employs *post-acute sequelae of SARS CoV-2 infection*. The heterogenous terminology, combined with the different definition criteria stated above, risks jingle-jangle fallacies. These fallacies can arise from falsely assuming two distinct syndromes are the same due to identical names (stemming from different definitions) or incorrectly assuming two distinct syndromes due to different names for the same phenomenon (resulting from varied terminology) [59].

These disparities in PCS definitions and terminology pose challenges to compare prevalence estimates, risk factors, and treatment effectiveness. Consequently, efforts should be directed towards enhancing clarity and comparability in future research.

The most common definitions in the included studies (WHO and NICE definitions) require a follow-up time of 12 weeks or longer. However, in the previous review by Lemhöfer et al., only 58% of all studies applied this time criterion. In the present review, this adherence rate was even lower with only 30% of all studies applying this criterion. Additionally, 24% of the studies did not employ any temporal inclusion criteria. This rate is marginally lower, but comparable to the findings of Lemhöfer et al., where 5 out of 16 studies (31%) did not implement any temporal inclusion criteria, and to a more recent review by Lai et al., which reported 25% without temporal inclusion criteria [55]. However, all included studies reported their results in relation to post- or long-COVID. Although there have been some improvements in the definitions of PCS in the included research since the publication of Lemhöfer et al., there still seems to be no clear consensus on the definition and temporal dimension of the syndrome.

Besides the benefit of enabling more comparable diagnoses of PCS, such temporal criteria could also help in precisely distinguishing the terminology for different COVID-19 sequelae, which are currently not sufficiently distinguished (see above). The currently revised S1 guideline on long/post-COVID by the German Society for Pneumology and Respiratory Medicine [60] suggests classifying symptoms persisting for more than four weeks after infection as ‘long COVID’ or ‘post-acute sequelae of COVID-19,’ and if they persist for more than twelve weeks, they should be referred to as ‘post-COVID syndrome’. Distinguishing between the criteria of 4–12 weeks and more than 12 weeks not only brings clarity to the definition of the conditions but also might have clinical relevance. Symptom prevalence evolves significantly over time; for example, the prevalence of loss of taste or smell, coughing, or diarrhea tends to decrease over several months, while the prevalence of dyspnea or word-finding problems remains stable, and the prevalence of pain or paraesthesia increases [61]. This leads to potentially significantly different clinical manifestations of post-COVID sequelae depending on the predominant symptoms over time, calling for different therapeutic interventions.

The variations in defining PCS outlined above may suggest persisting challenges in its diagnosis. Notably, the most commonly used definitions by the WHO and the NICE require symptoms after a SARS-CoV-2 infection that are not explained by another alternative diagnosis [11, 16]. This could pose challenges for clinicians and researchers, as it is often difficult to differentiate whether symptoms already existed before the SARS-CoV-2 infection [62], whether they were exacerbated but not caused by the infection [63, 64], or whether consequences such as sleep disturbances or impairments in physical activity were caused by pandemic response measures [65, 66], but not by the infection itself. Often, there are no baseline data on the patients and it is therefore difficult to differentiate which symptoms occurred as a result of the infection and which were already present [12]. By relying on broader definitions such as the PCS definitions of the NIH or the CDC or by a self-developed definition, which only require the presence of symptoms after a certain follow-up period after infection, it might be easier for researchers to diagnose PCS because no alternative explanations have to be ruled out. However, this also leads to more false-positive and thus an overestimation of prevalence. Additionally, most definitions of PCS require some form of confirmation of a SARS-CoV-2 infection. The majority of the included studies addressed the necessity of confirming SARS-CoV-2 infection for properly diagnosing PCS. The most common method of infection confirmation was PCR testing, followed by antibody tests. However, nearly a third of the studies did not address this requirement. To further increase the reliability of PCS diagnosis, it might be beneficial to reach a consensus on the methods for confirming SARS-CoV-2 infection.In their review, Lemhöfer et al. also criticize the lack of consensus on the symptoms required to diagnose PCS. While they do not offer an exhaustive overview of the symptoms of PCS as reported in their included studies, they indicate that fatigue and cognitive impairments are the most commonly reported symptoms. In our updated review, we find that fatigue was indeed the symptom reported in all but one study, and neurological (cognitive) impairments were reported by more than four out of five studies, followed by exercise intolerance. This representation of symptoms in the literature reflects findings of high prevalence of these symptoms among COVID-survivors [14]. Other symptoms reported in the majority of studies were joint and/or muscle pain, psychiatric symptoms, sleep disturbances, cardiac symptom, pulmonary symptoms, chemosensory deficits, ear-nose-throat ailments, and gastrointestinal ailments. Symptoms such as infection signs and dermatological ailments are only reported in the minority of studies and appear to be less relevant in the diagnosis of PCS. However, they may still be beneficial to assess functional assessments, reduced quality of life, or to identify patient subgroups. Renal symptoms, or urinary symptoms were the least reported across all studies. Overall, the results of this umbrella review show that the current focus of research on PCS symptoms seems to represent the actual prevalence of symptoms among COVID survivors. This is also reflected in a recently developed standardized assessment of PCS severity by Bahmer at al. [13]. The authors developed a PCS score, that is defined by weighted symptoms. Although these weights do not necessarily reflect symptom frequency but rather symptom severity, they are generally well aligned with the symptoms reported in this study. However, psychiatric symptoms in relation to the PCS were reported by more than two thirds of the included studies. Although the PCS score includes neurological symptoms such as confusion, vertigo, headache, motor deficits, sensory deficits, numbness, tremor, deficits of concentration, cognition or speech, it does not currently include psychiatric symptoms such as anxiety or depressive mood. However, with regard to the results reported here, these symptoms should be taken into account when assessing PCS severity, as there is a rather high prevalence of psychiatric disorders among COVID survivors, especially of depression (prevalence of 3–65%) and anxiety (prevalence of 16 − 48%) [22, 67–70].

Our umbrella review reveals a meaningful shift in the research on PCS-related QOL and functional impairments. A narrow majority of 55% of all studies now reports on PCS-related QOL, a marked increase from the mere 13% reported by Lemhöfer et al. [4]. This increase found in the present study contrasts with a recent review analyzing clinical trials of adult PCS patients, which reported only 24% of studies (15/63) addressing QOL [71]. However, less than half of the studies in our analysis explicitly state which assessment tools that were employed. The most commonly utilized questionnaire was the SF-36 [72], which is a well-established instrument assessing vitality, physical functioning, bodily pain, general health perceptions, role limitations due to physical health and emotional health, social role functioning and mental health. Alongside the increased reporting on QOL, 71% of the included studies assessed functional impairments, far exceeding the 13% in Lemhöfer et al.’s findings and 16% of studies reported in a recent review [71]. The most frequently reported issue was cognitive impairments, yet only a third of the included studies addressed this aspect. These findings are in concordance with the emerging consensus that QOL and everyday functioning are critical in the diagnosis and treatment of PCS [12, 13]. Nonetheless, akin to the above reported lacking consensus on PCS definition and its requisite symptoms, there remains considerable variability in the assessment of these impairments and determining which aspects of QOL and functional impairment warrant the most attention. Taken together, it is imperative that both practitioners and researchers employ standardized tools to assess QOL and functional impairments. The former might be assessed with the SF-36 as this was the most frequently reported assessment tool. The latter might be assessed with, for example, questionnaires based on the International Classification of Functioning, Disability and Health (ICF) [73]. This is particularly important as the rehabilitation of patients, the restoration of their ability to work and the maintaining and resuming of activities of daily living play a vital role in the treatment of PCS [74–77]. Additionally, the present updated review confirms the need of further research on vulnerable populations. Almost 40% of the studies reported here did not include older adults above 60 years of age, and, most importantly, no studies reported presence of frailty in the older patients studied. In light of the demographic transition and the increasing relevance of multidimensional frailty as a proxy of biological age, an accurate clinical description of older study participants may also help disclosing disease presentation and better treatment in advanced age [78]. Finally, this review focuses on PCS definitions in current research. However, we did not analyze which definitions are used in different national guidelines published by health organizations around the world. In future research, a thorough analysis that systematically screens such guidelines for their use of WHO, NICE, CDC, or NIH definitions in their long/post-COVID guidelines would be highly beneficial. Such an investigation could provide valuable insights into variations in international guideline adherence and uncover different local recommendations that influence these practices.

## Conclusion

In conclusion, research on PCS has increased substantially in both quantity and significance attributed to functional impairments since the publication of Lemhöfer et al. in 2022. While progress has been made in clarifying the definition of PCS, achieving consensus on the symptoms essential for a definitive diagnosis remains elusive. Many researchers advocate for the inclusion of psychiatric symptoms, QOL, frailty and functional impairments in these considerations, highlighting their importance in understanding and managing PCS comprehensively. The present umbrella review is crucial in providing a comprehensive analysis of PCS definitions and associated symptoms, which is essential for improving diagnostic criteria and research methodologies. By highlighting the gaps and inconsistencies in current PCS research, our study aims to guide future investigations, ultimately enhancing the understanding and management of post-COVID syndrome.

## Electronic supplementary material

Below is the link to the electronic supplementary material.

Supplementary Material 1
